# Source control within 12 h attenuates lung injury and systemic bacterial burden in a rat model of polymicrobial abdominal sepsis (cecal ligation and puncture)

**DOI:** 10.1186/s40635-026-00941-1

**Published:** 2026-07-17

**Authors:** Maria Luisa Martinez, Eva Torrents, Marta Camprubí-Rimblas, Josep Bringué, Adrian Ceccato, Ricard Ferrer, Antonio Artigas, Raquel Guillamat-Prats

**Affiliations:** 1 Critical Care Research Center, Parc Taulí Hospital Universitari, Institut d’Investigació I Innovació Parc Taulí (I3PT-CERCA), Universitat Autònoma de Barcelona, Sabadell, Spain; 2 CIBER de Enfermedades Respiratorias (CIBERES), Sabadell, Spain; 3 Department of Critical Care, Parc Taulí Hospital Universitari. Corporació Sanitària I Universitària Parc Taulí, Sabadell, Spain; 4 Department of Critical Care, Hospital Universitario General de Catalunya, Barcelona, Spain; 5 Department of Intensive Care, Vall d’Hebron University Hospital, Barcelona, Spain; 6 SODIR, Vall d’Hebron Institut de Recerca, Barcelona, Spain; 7 Respiratory and Immune Repair (REPAIR), Germans Trias I Pujol Research Institute (IGTP), Badalona, Spain

**Keywords:** Sepsis, Source control, Cecal ligation and puncture, Inflammation, Lung injury, Experimental model

## Abstract

**Background:**

Early and adequate source control (SC) is a cornerstone of sepsis management, yet experimental data linking delays in SC to bacterial dissemination and remote organ injury remain limited. We aimed to determine how the timing of surgical SC after polymicrobial abdominal sepsis influences bacterial burden, host inflammatory response, and lung injury. Hemodynamic recovery was assessed as a secondary outcome, and survival as an exploratory outcome.

**Results:**

Adult Wistar rats underwent cecal ligation and puncture (CLP) and received standardized fluid resuscitation and meropenem. Surgical SC (cecal resection plus peritoneal lavage) was performed at 6, 12, 18 or 24 h after CLP, or not performed, and outcomes were assessed at 72 h (hemodynamics, lactate, bacterial counts in blood/peritoneal lavage/bronchoalveolar lavage, systemic and pulmonary cytokines, bronchoalveolar lavage cellularity and histology). Surgical SC within 6–12 h was associated with lower bacterial burden across blood, peritoneal lavage, and bronchoalveolar lavage, together with attenuation of systemic and pulmonary inflammatory mediators and reduced lung injury. These biological effects were accompanied by improved mean arterial pressure, lower lactate, and preservation of body weight. Survival analysis suggested a numerically time-dependent pattern according to SC timing; however, these data were exploratory and the study was not powered to detect mortality differences.

**Conclusions:**

In this clinically relevant CLP rat model, earlier surgical SC (≤ 12 h) was associated with lower bacterial burden, attenuation of systemic and pulmonary inflammation, reduced sepsis-associated lung injury, and improved physiological recovery. These findings reinforce the importance of timely source control in abdominal sepsis and provide experimental support for avoiding unnecessary delay.

**Supplementary Information:**

The online version contains supplementary material available at 10.1186/s40635-026-00941-1.

## Background

Sepsis is a life-threatening syndrome caused by a dysregulated host response to infection leading to organ dysfunction [1]. Despite advances in sepsis pathophysiology, management, development of novel antimicrobials and standardized supportive care protocols, it remains a major global health challenge responsible for over 11 million deaths annually [2, 3]. Early diagnosis, appropriate antibiotics, and effective source control (SC) constitute the essential triad of sepsis management [4].

SC encompasses all physical interventions to eliminate the infectious focus, reduce microbial load, and restore anatomy and function [5]. It includes drainage, debridement, decompression, and definitive repair. Rapid pathogen clearance is a central determinant of outcomes in septic patients [6], and clinical evidence consistently demonstrates that inadequate or delayed SC is associated with increased mortality [7, 8]. The primary goal of SC in sepsis is to eliminate or reduce the bacterial burden at the site of infection, thereby removing the main trigger of the host’s inflammatory response and subsequent organ dysfunction. Nevertheless, defining the “golden window” for intervention remains difficult because of heterogeneity in infection sites, host responses, and logistical constraints [9]. The 2026 Surviving Sepsis Campaign guidelines recommend performing any required SC “as soon as medically and logistically practical” [4], ideally in the first 6 h. However, the optimal timeframe is unknown. Quantitative experimental data delineating the biological impact of delayed intervention remain scarce.

Animal models are indispensable for mechanistic insight. Among them, cecal ligation and puncture (CLP), the gold standard approach, reproduces the polymicrobial peritonitis, hemodynamic instability, and cytokine storm typical of human abdominal sepsis [10, 11]. However, most CLP studies omit definitive SC, focusing instead on antibiotic or immunologic interventions. Integrating timed SC into the CLP model provides a unique opportunity to simulate clinical decision-making and assess how delays alter bacterial clearance, systemic inflammation, and distal organ injury, particularly in the lungs, which are highly susceptible to sepsis-induced damage [12].

Translational data from patients suggest that every hour of delay in controlling the infectious focus may increase mortality risk [13, 14]. Yet, the molecular and physiological mechanisms underpinning this relationship remain poorly characterized. Understanding these mechanisms in experimental systems may help refine the timing of source control in clinical practice.

Therefore, the present study aimed to determine how the timing of surgical source control after CLP influences bacterial burden, inflammatory responses, and lung injury in a well-characterized rat model of abdominal sepsis. We hypothesized that earlier source control would improve microbial containment by removing the infectious focus before it could seed distant compartments, thereby limiting bacterial dissemination. We further hypothesized that lower bacterial burden would be associated with reduced systemic and pulmonary inflammatory responses and less remote organ injury, particularly in the lungs. Hemodynamic recovery was assessed as a secondary outcome, whereas survival was included as an exploratory outcome.

## Methods

### Ethical approval and animal care

All experimental procedures were approved by the Animal Ethics Committee of the Autonomous University of Barcelona (UAB) and the Animal Experimentation Committee of the Generalitat de Catalunya (approval number CEAAH 2407). The study was conducted in accordance with Directive 2010/63/EU on the protection of animals used for scientific purposes.

Adult male Wistar rats (*Rattus norvegicus*), weighing 200–250 g and aged 8–9 weeks, were obtained from Charles River Laboratories (Spain). Animals were housed under controlled temperature (22 ± 1 °C) and humidity (55 ± 5%) conditions with a 12-h light/dark cycle and provided food and water ad libitum.

### Experimental model of sepsis

#### Cecal Ligation and Puncture (CLP)

Abdominal sepsis was induced using the well-established CLP [10, 11, 15]. Rats were anesthetized by intraperitoneal injection of ketamine (80–100 mg/kg) and xylazine (10–12 mg/kg). Following hair removal and antisepsis with povidone–iodine and alcohol, a 2-cm midline laparotomy was performed to expose the cecum.

The cecum was ligated just below the ileocecal valve to avoid intestinal obstruction, and a second ligature was placed approximately 1 cm from the cecal tip. The segment between the ligatures was punctured four times with an 18-gauge needle: once near the distal end and three times between ligatures, ensuring consistent leakage of fecal content. The bowel was repositioned, and the abdomen closed in two layers using monofilament sutures.

#### Source control

SC consisted of reopening the laparotomy at the specified interval, debriding the necrotic cecal segment, and performing peritoneal lavage with three washes of pre-warmed 0.9% saline solution. The abdomen was closed in two layers. The selected time points (6, 12, 18, and 24 h after CLP) were chosen to model clinically relevant delays in source control across the transition from early to established abdominal sepsis. This design was intended to capture progressive differences in bacterial dissemination, inflammatory responses, and remote organ injury as infection evolved over time. All animals received broad-spectrum antibiotic therapy with meropenem (20 mg/kg subcutaneously) starting at 6 h after CLP and every 12 h thereafter until sacrifice at 72 h. Because antibiotics were initiated identically in all CLP groups, between-group differences in bacterial and inflammatory outcomes should be interpreted as reflecting the combined effects of source elimination and antibiotic exposure.

#### Experimental groups

Animals were randomly assigned to one of six groups:

1. Sham (n = 13): laparotomy with exteriorization of the cecum but without ligation or puncture.
2. CLP (n = 12): sepsis induced by CLP without subsequent surgical intervention.
3. CLP + SC-6 h (n = 13): surgical source control performed 6 h after CLP.
4. CLP + SC-12 h (n = 13): surgical source control performed 12 h after CLP.
5. CLP + SC-18 h (n = 14): surgical source control performed 18 h after CLP.
6. CLP + SC-24 h (n = 15): surgical source control performed 24 h after CLP.

For supplementary exploratory analyses, SC groups were also pooled as early SC (6 h + 12 h) and delayed SC (18 h + 24 h).

#### Resuscitation and supportive therapy

Fluid resuscitation with isotonic saline (35 mL/kg, 37ºC) was administered subcutaneously immediately after laparotomy and repeated at 6 h and 18 h. Analgesia was maintained with buprenorphine (0.05 mg/kg every 12 h). Rats were monitored for activity, grooming, and appearance at regular intervals.

#### Sampling and euthanasia

All surviving animals were euthanized at 72 h after CLP by exsanguination under deep anesthesia. Whole blood was collected from the abdominal aorta into EDTA tubes for microbiological culture and plasma separation.

#### Hemodynamic and physiological measurements

Body weight (g) was recorded before surgery and at 72 h. Mean arterial pressure (MAP), arterial carbon dioxide (PaCO₂), oxygen saturation (SatO₂), and lactate concentrations were measured 72 h after CLP. MAP was obtained noninvasively by tail-cuff volume pressure recording (CODA system, Kent Scientific), and arterial blood gases were analyzed with a Dri-Tek BG-OX Cartridge biochemical analyzer.

#### Bacterial culture and quantification

Quantitative bacterial cultures were performed from blood, peritoneal lavage, and BAL fluids using serial dilution plating.

- Blood samples: 100 µL total volume, 50 µL undiluted and 50 µL diluted 1:10 in phosphate-buffered saline (PBS), plated on 5% sheep blood agar (Fisher Scientific, Pittsburgh, PA).
- Peritoneal lavage and BAL: 100 µL samples diluted 1:10 and cultured on 5% sheep blood agar.

Plates were incubated at 37 °C for 24–48 h under both aerobic and anaerobic conditions. Colonies were counted to determine colony-forming units (CFU/µL).

Microbial identification was performed using the VITEK® 2 automated system (bioMérieux, France), following growth-based biochemical identification protocols (VITEK® 2 User’s Manual, bioMérieux).

### Protein and inflammatory mediator measurements

#### Measurement of total protein concentration

Protein concentration in plasma, peritoneal lavage and BAL supernatant was measured using the bicinchoninic acid (BCA) assay (Thermo Fisher Scientific Inc., Waltham, Massachusetts).

#### Plasma cytokines (ELISA)

Plasma TNF-α and IL-6 levels were quantified using commercial enzyme-linked immunosorbent assay (ELISA) kits (R&D Systems, Minneapolis, MN), according to the manufacturer’s instructions. Briefly, capture antibody was coated on 96-well plates overnight, followed by blocking, incubation with samples and standards (2 h, room temperature), detection antibody incubation (2 h), and reaction development with streptavidin–horseradish peroxidase (HRP) and tetramethylbenzidine (TMB) substrate. The reaction was stopped with 0.1 M HCl, and optical density was read at 450 nm. Cytokine concentrations were interpolated from standard curves.

#### Lung cytokines (multiplex assay)

Lung homogenates were prepared in lysis buffer containing 25 mM Tris–HCl (pH 7.6), 150 mM NaCl, 1% NP-40, 1% sodium deoxycholate, 0.1% SDS, 1 mM sodium orthovanadate, and protease inhibitor cocktail (Roche, Mannheim, Germany). Homogenates were centrifuged (12,000 rpm, 20 min, 4 °C), and the supernatants were used for cytokine determination using Luminex xMAP® technology (Affymetrix, Fremont, CA), quantifying tumor necrosis factor-alpha (TNF-α), interleukin-6 (IL-6), interleukin-1 beta (IL-1β), interleukin-2 (IL-2), interleukin-4 (IL-4), interferon-gamma (IFN-γ), growth-regulated oncogene-alpha (GRO-α, also known as CXCL1), granulocyte–macrophage colony-stimulating factor (GM-CSF), monocyte chemoattractant protein-1 (MCP-1, also known as CCL2), interleukin-10 (IL-10), and interleukin-13 (IL-13). This panel was selected a priori based on its relevance to sepsis-induced pulmonary inflammation in the CLP literature and the availability of validated rat multiplex assays, with emphasis on mediators involved in neutrophil recruitment, macrophage activation, and pro-/anti-inflammatory balance.

#### BAL cell counts and lung injury score

Total cell counts in BAL fluid were determined by flow cytometry. Neutrophils were identified as CD11b⁺/Ly6G⁺ populations. Fixed lungs were embedded in paraffin, sectioned (4 µm), and stained with hematoxylin and eosin (H&E). Lung injury was scored semi-quantitatively by two blinded investigators, assessing: alveolar hemorrhage, neutrophil infiltration, and alveolar wall thickening or collapse. Each parameter was graded on a scale from 0 (none) to 3 (severe), and total lung injury score (LIS) was the sum of these components (maximum = 9).

### Statistical analysis

Statistical analyses were performed with GraphPad Prism v9.0 (GraphPad Software, San Diego, CA). The primary endpoints were bacterial burden, systemic and pulmonary inflammatory responses, and lung injury. Hemodynamic and metabolic variables were assessed as secondary outcomes. Sample size was determined a priori using G*Power (version 3.1) based on expected differences in bacterial CFU counts derived from published CLP data and our laboratory’s prior experience with this model, targeting 80% power at a two-tailed α of 0.05. Initial group sizes were set at 12–15 animals to ensure that sufficient survivors would be available for biochemical and histological endpoints at 72 h after anticipated mortality. Outliers were identified using the ROUT test (Q = 1%). Normality was assessed by the D’Agostino–Pearson omnibus test. Comparisons between two groups were made using the unpaired Student’s t-test for normally distributed data or the Mann–Whitney U-test for non-normal distributions. For comparisons across ≥ 3 groups, one-way or two-way ANOVA followed by Bonferroni’s post hoc test was used. For multiplex cytokine data, each mediator was analyzed independently by one-way ANOVA with Bonferroni post hoc correction; no additional adjustment for the number of cytokines measured was applied, and these results should be interpreted in an exploratory context. Survival data were analyzed using Cox proportional-hazards modeling with Firth’s penalized likelihood correction, with the CLP group used as the reference category. Survival curves were also compared using the log-rank (Mantel–Cox) test. In supplementary exploratory analyses, pooled early SC (6 h + 12 h) was compared with pooled delayed SC (18 h + 24 h). Data are presented as mean ± SD. A two-tailed p < 0.05 was considered statistically significant.

## Results

### Body weight and survival

**Fig. 1:**
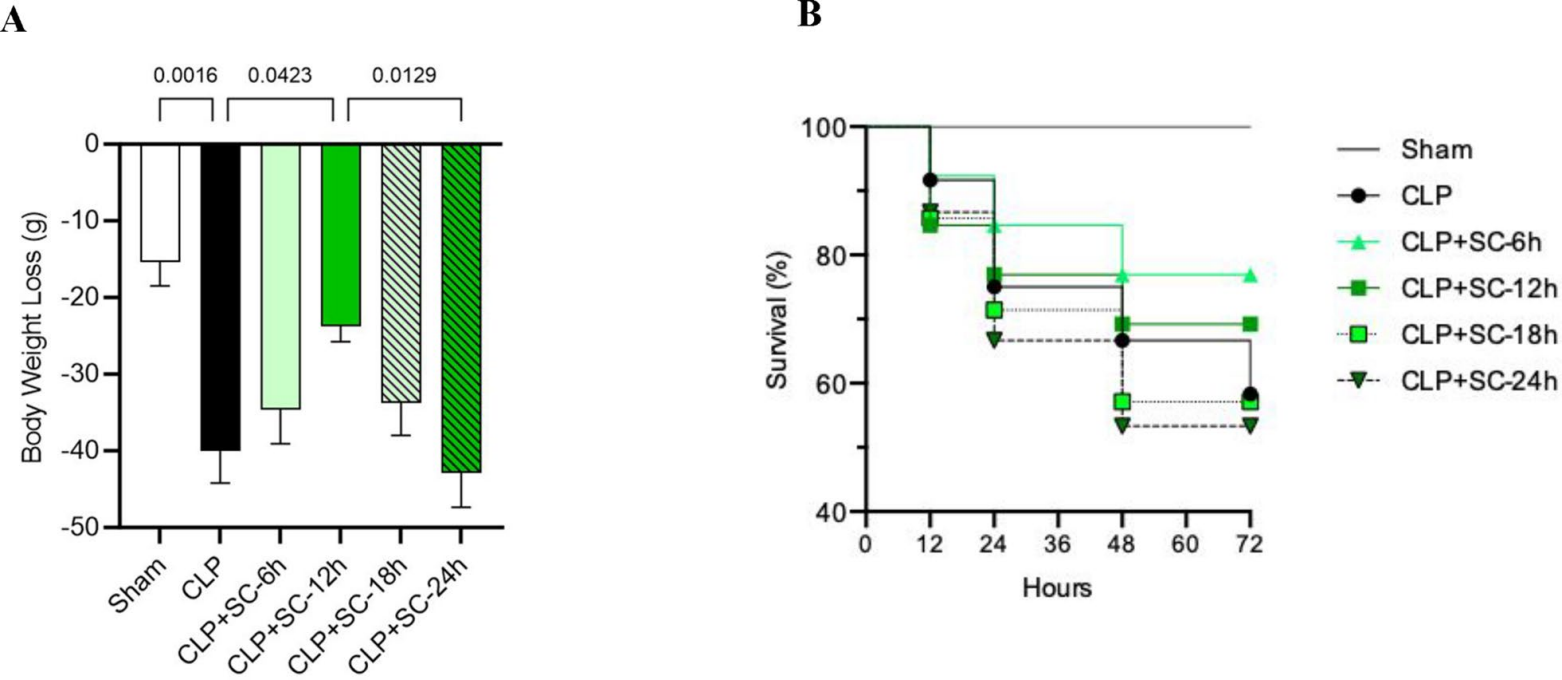
Body weight and survival. (**A**) Body weight loss at 72 h relative to baseline body weight in all groups. Data are presented as mean ± SD. At 72 h, the numbers of animals available for endpoint analyses were 13 (Sham), 7 (CLP), 10 (CLP + SC-6 h), 9 (CLP + SC-12 h), 8 (CLP + SC-18 h), and 8 (CLP + SC-24 h). (**B**) Kaplan–Meier survival curves over the 72-h experimental period. Numbers included in the survival analysis were 13 (Sham), 12 (CLP), 13 (CLP + SC-6 h), 13 (CLP + SC-12 h), 14 (CLP + SC-18 h), and 15 (CLP + SC-24 h). Deaths/events were 0, 5, 3, 4, 6, and 7, respectively, and numbers censored at 72 h were 13, 7, 10, 9, 8, and 8, respectively. Median survival was not reached in any group

All animals experienced postoperative weight loss following sham or CLP procedures (Fig. 1A). Animals undergoing early SC (6–12 h) demonstrated significantly attenuated weight loss compared with both the CLP group and delayed SC (18–24 h) groups (p < 0.05) (Fig. 1A and Additional file 1: Supplementary Fig. 1A).

Survival analysis at 72 h did not show statistically significant between-group differences according to SC timing (Fig. 1B and Additional file 1: Supplementary Fig. 1B), and should be considered exploratory because the study was not powered to detect mortality differences. In the Cox proportional-hazards model with Firth’s penalized correction, using the CLP group as the reference category, hazard ratios were 0.57 (95% CI 0.14–2.38) for CLP + SC-6 h, 0.75 (0.20–2.79) for CLP + SC-12 h, 1.09 (0.33–3.59) for CLP + SC-18 h, and 1.22 (0.39–3.86) for CLP + SC-24 h. Overall, between-group differences were not statistically significant (global p = 0.393). Deaths occurred across all CLP groups beginning as early as 12 h post-CLP (Fig. 1B), and some animals assigned to the 18 h and 24 h SC groups may therefore not have survived long enough to receive the planned intervention. A Cox regression summary (coefficients, hazard ratios, and 95% confidence intervals) is provided in Table 1.

**Table 1:** Cox regression summary of 72‑hour survival by source‑control timing

Intervention	Coefficient (coef)	Hazard ratio (HR)	95% CI (lower – upper)	p‑value
*CLP (reference)*	0.000	1.00	1.00 – 1.00	—
CLP + SC‑6 h	–0.564	0.57	0.14 – 2.38	0.440
CLP + SC‑12 h	–0.291	0.75	0.20 – 2.79	0.665
CLP + SC‑18 h	0.090	1.09	0.33 – 3.59	0.882
CLP + SC‑24 h	0.203	1.22	0.39 – 3.86	0.729

CLP, cecal ligation and puncture; SC, source control; coef, regression coefficient of the Cox model; HR, hazard ratio; CI, confidence interval. An HR > 1 indicates increased hazard (reduced survival), whereas an HR < 1 indicates decreased hazard (improved survival), relative to the CLP group.

### Hemodynamic parameters and organ dysfunction

**Fig. 2:**
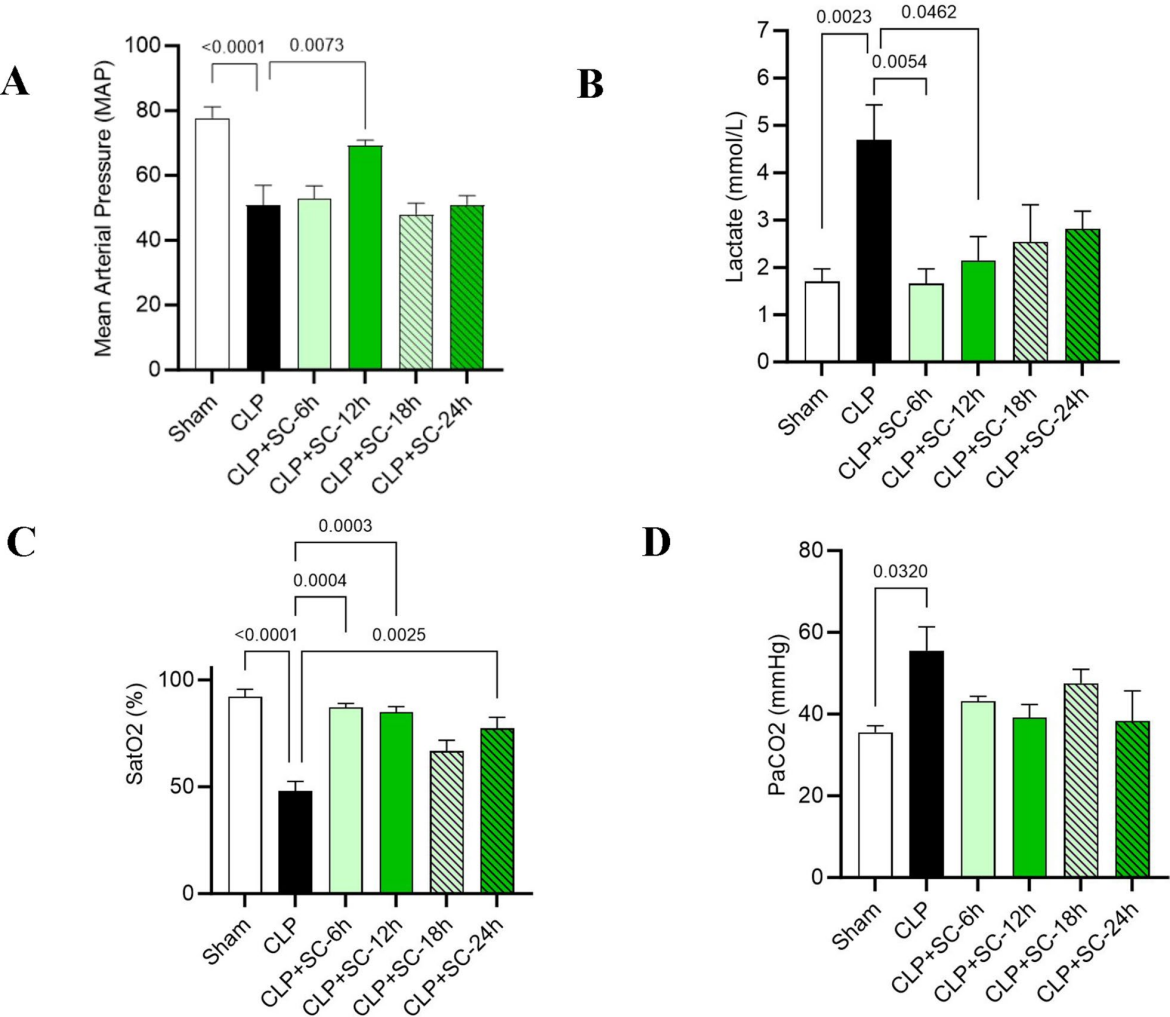
Hemodynamic and physiologic variables. (**A**) Mean arterial pressure (MAP). (**B**) Serum lactate concentration. (**C**) Oxygen saturation (SatO2). (**D**) Arterial partial pressure of carbon dioxide (PaCO2). Values are presented as mean ± SD. At 72 h, n = 7–13 animals per group were available for endpoint analyses

At 72 h post-CLP, CLP animals presented sustained hypotension (MAP = 51 ± 4 mmHg) compared with sham controls (78 ± 3 mmHg, p < 0.0001). Among SC groups, only CLP + SC-12 h showed a significantly higher MAP compared with CLP (63.3 ± 3.5 mmHg vs. 51 ± 4 mmHg, p < 0.01; Fig. 2A), whereas the CLP + SC-6 h group did not differ significantly from CLP in MAP. Plasma lactate levels were significantly higher in the CLP group (4.7 ± 0.71 mmol/L) compared to sham animals (1.7 ± 0.32 mmol/L, p < 0.01). Both CLP + SC-6 h and CLP + SC-12 h showed significant reductions in lactate compared with CLP (p < 0.05; Fig. 2B; Additional file 2: Supplementary Fig. 2A).

Arterial blood gas analysis at 72 h revealed significant hypoxemia and hypercapnia in the CLP group, consistent with sepsis-induced lung dysfunction.19 Both early SC groups (CLP + SC-6 h and CLP + SC-12 h) showed significantly improved SatO₂ compared with CLP (p < 0.01; Fig. 2C), and in the pooled comparison, early SC was associated with significantly higher SatO₂ than delayed SC (p = 0.0020; Additional file 2: Supplementary Fig. 2B). PaCO₂ showed a numerical reduction in SC groups compared with CLP, but differences did not reach statistical significance in either individual-group or pooled comparisons (Fig. 2D; Additional file 2: Supplementary Fig. 2C).

### Bacterial burden

**Fig. 3:**
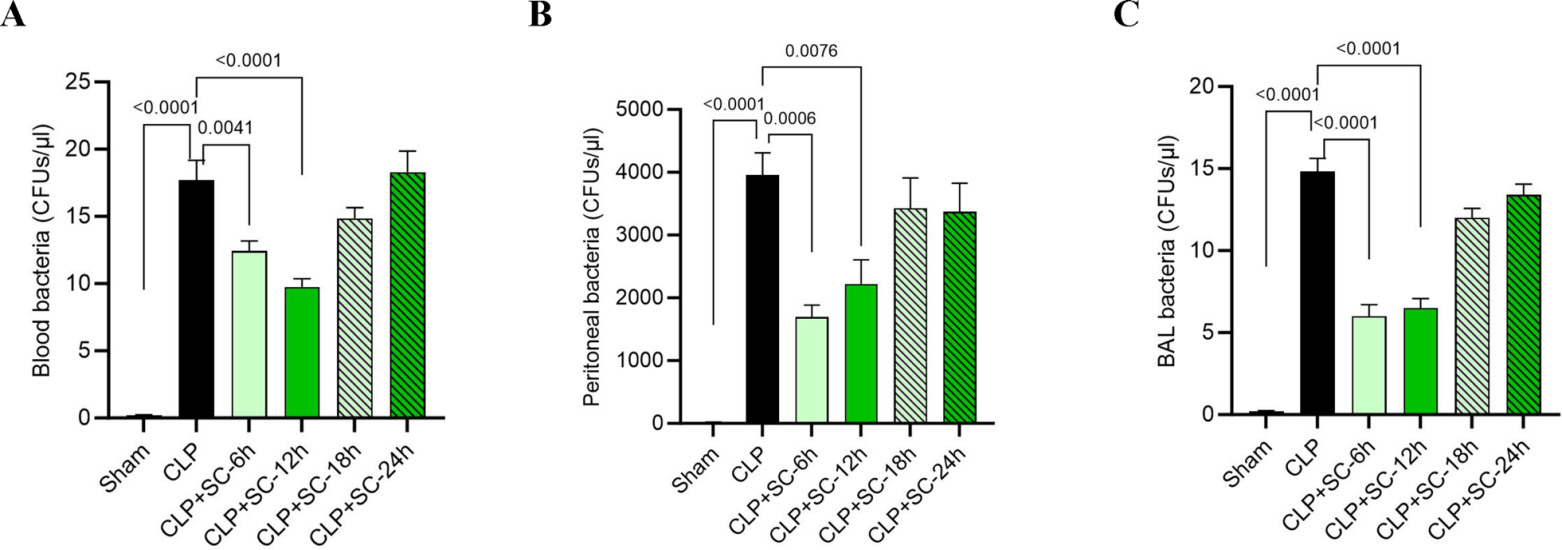
Bacterial counts. (**A**) Blood bacterial counts. (**B**) Peritoneal bacterial counts. (**C**) Bronchoalveolar lavage (BAL) bacterial counts. Values are presented as mean ± SD. At 72 h, n = 7–13 animals per group were available for endpoint analyses

Quantitative cultures confirmed polymicrobial infection dominated by Gram-negative species (*Escherichia coli, Proteus mirabilis, Bacteroides fragilis, Bacteroides* spp.). At 72 h, bacterial counts were markedly elevated in blood, peritoneal lavage, and BAL fluids in the CLP group compared with sham controls (p < 0.001; Fig. 3A–C). Early SC (6–12 h) significantly reduced CFU counts across all compartments (p < 0.01), while delayed SC (18–24 h) failed to achieve comparable clearance (Additional file 2: Supplementary Fig. 2D-F).

**Fig. 4:**
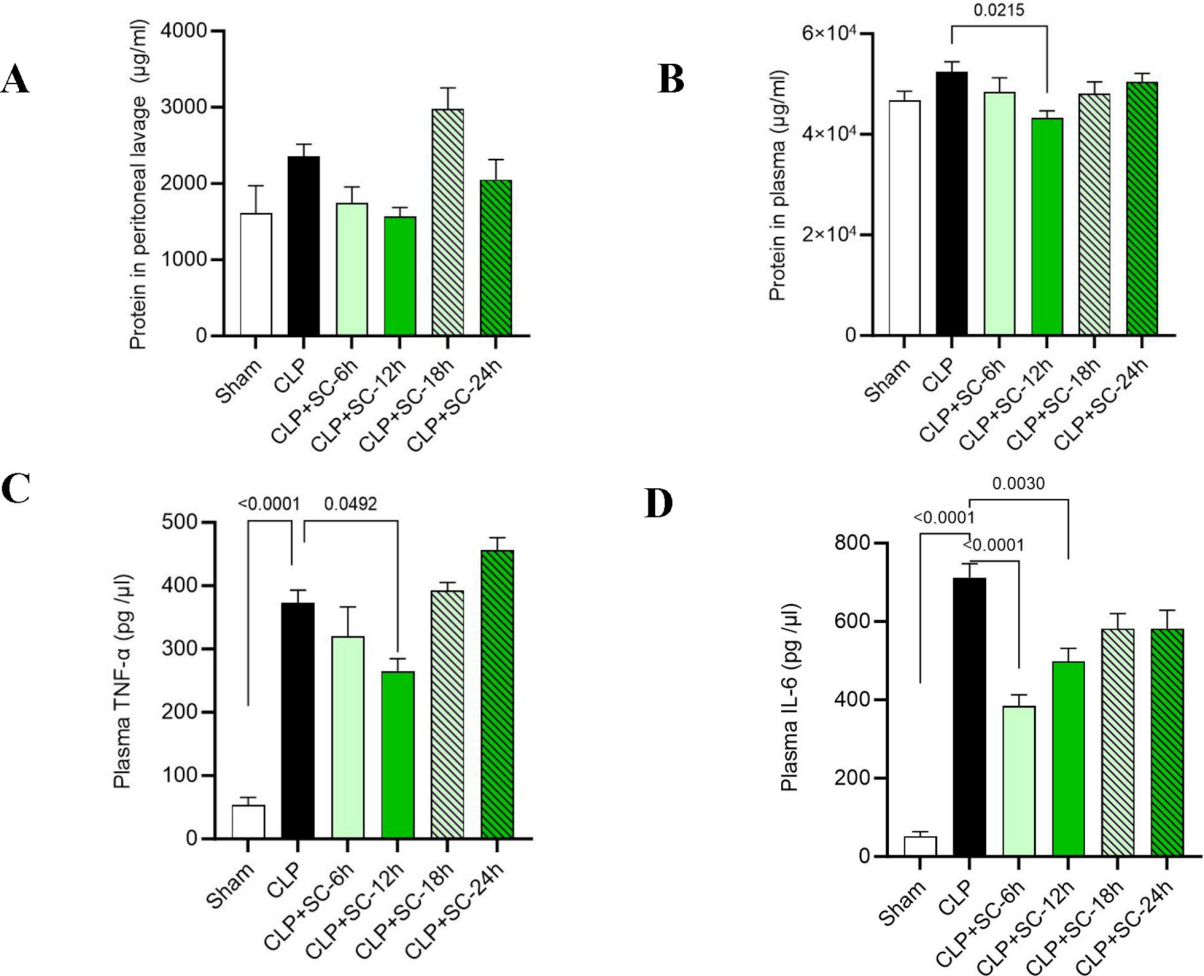
Peritoneal and plasma inflammatory markers. (**A**) Total protein concentration (μg/mL) in peritoneal lavage. (**B**) Total protein concentration (μg/mL) in plasma. (**C**) Plasma tumor necrosis factor-α (TNF-α). (**D**) Plasma interleukin-6 (IL-6). Values are presented as mean ± SD. At the 72-h time point, 7–8 animals per group were available for these analyses. The number of samples analyzed was lower than the total number of surviving animals because insufficient plasma or peritoneal lavage volume remained in some samples

Peritoneal lavage protein concentrations were elevated following CLP and further increased in the group receiving SC at 18 h; no significant differences were observed among the other groups (Fig. 4A). In contrast, plasma protein concentrations were significantly lower in the CLP + SC-12 h group compared to the CLP group (p < 0.05; Fig. 4B).

### Systemic inflammatory response (cytokine levels)

Plasma concentrations of TNF-α and IL-6, two key pro-inflammatory cytokines, were markedly elevated in septic rats without SC compared with sham controls (Fig. 4C-D). This increase was attenuated after SC, with the clearest reductions observed in the early intervention groups (Fig. 4C-D and Additional file 2: Supplementary Fig. 2G-H).

### Lung inflammation

**Fig. 5:**
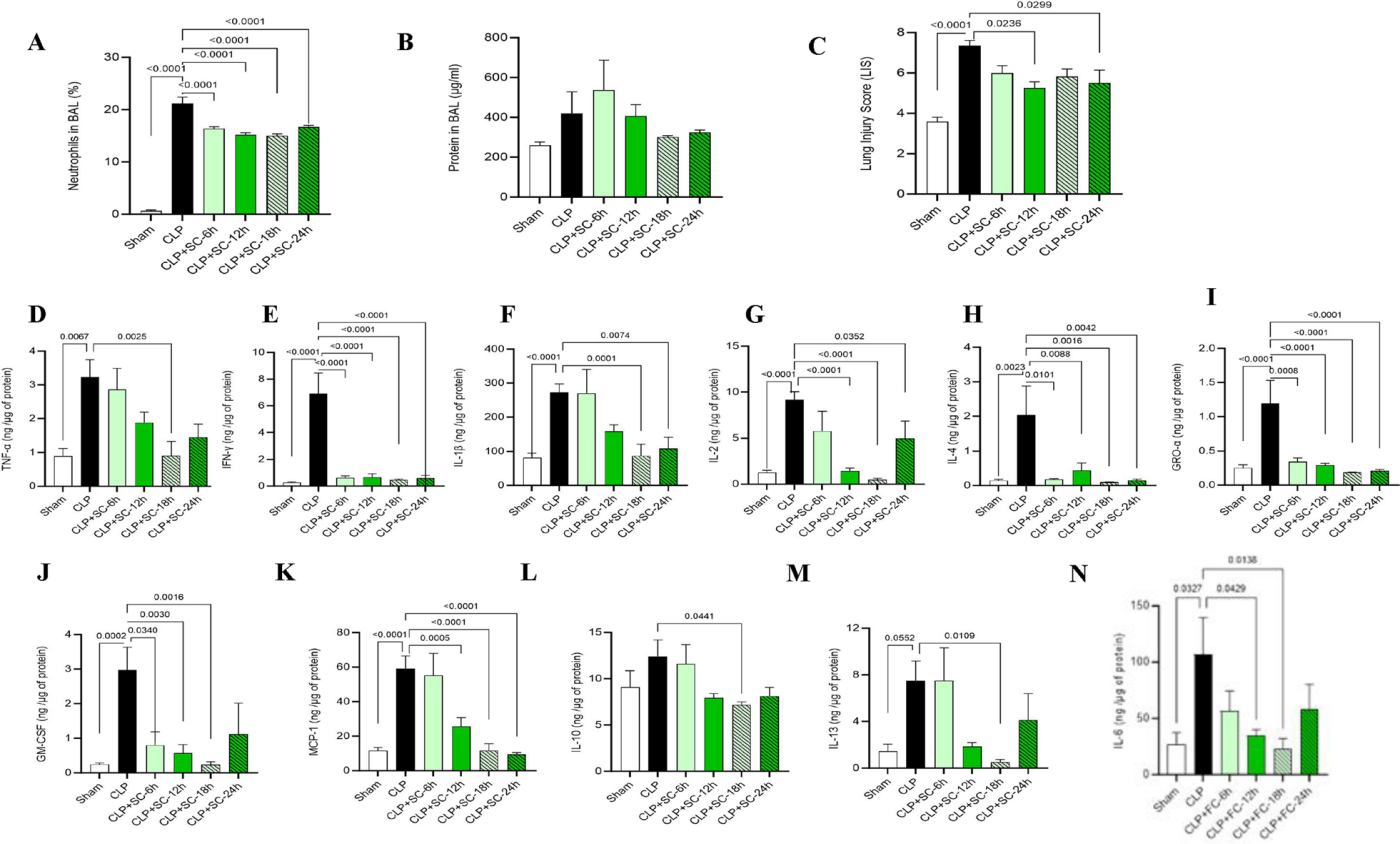
Lung injury parameters. (**A**) Percentage of neutrophils in bronchoalveolar lavage (BAL). (**B**) Total protein concentration in BAL. (**C**) Lung injury score (LIS) evaluated in H&E-stained lung sections. Lung homogenate cytokines were measured by multiplex assay: (**D**) TNF-α, (**E**) IFN-γ, (**F**) IL-1β, (**G**) IL-2, (**H**) IL-4, (**I**) GRO-α, (**J**) GM-CSF, (**K**) MCP-1, (**L**) IL-10, (**M**) IL-13, and (**N**) IL-6. Values are presented as mean ± SD. At 72 h, 7–8 animals per group were available for these analyses

Flow cytometry of BAL samples demonstrated a significant influx of neutrophils in the CLP group, reaching 21.1 ± 1.2% of the total cell population. This neutrophilic infiltration was significantly attenuated in animals with SC (p < 0.0001; Fig. 5A and Additional file 3: Supplementary Fig. 3A). In contrast, total protein concentration in BAL fluid—a marker of alveolar-capillary permeability—showed no significant differences among the experimental groups (Fig. 5B).

Analysis of lung homogenates showed that SC broadly attenuated the pulmonary cytokine response, although the magnitude of reduction varied across mediators and time points of SC (Fig. 5D–N). Lung homogenate IL-6 was significantly increased in CLP compared with Sham (p = 0.0327), and was significantly reduced in the 12 h (p = 0.0429) and 18 h (p = 0.0138) SC groups relative to CLP, whereas the 6 h and 24 h groups were numerically lower than CLP but not significantly different. The 12 h SC group showed one of the clearest combined patterns of cytokine reduction, although this effect was not uniform across all biomarkers. In supplementary exploratory pooled early-versus-delayed comparisons, differences were also observed for neutrophils, lung injury score, and selected lung cytokines (Additional file 3: Supplementary Fig. 3). Both pooled early and pooled delayed SC were associated with reductions in several pro-inflammatory mediators relative to CLP, whereas IL-10 and IL-13 were lower in some delayed-SC comparisons (Additional file 3: Supplementary Fig. 3C–L).

### Histopathological findings

Lung histology confirmed that SC attenuated respiratory injury (Fig. 5C). CLP rats developed severe lung injury (LIS 7.3 ± 0.4) at 72 h. Early SC at 12 h was associated with the lowest injury score (LIS 5.2 ± 0.3; p < 0.05 vs. CLP in the main comparison), while delayed SC groups showed a more limited improvement. In the early-versus-late comparison, both pooled early and pooled delayed SC groups were associated with lower lung injury scores than CLP, whereas the difference between early and delayed intervention was less pronounced (Additional file 3: Supplementary Fig. 3B).

## Discussion

Our findings indicate that earlier surgical SC was associated with lower bacterial burden, a more favorable inflammatory profile, less lung injury, and better physiological recovery in this polymicrobial CLP model. By contrast, delaying SC beyond 18 h was associated with persistent bacteremia, sustained inflammation, and more severe pulmonary injury. Although the survival curves showed a numerical time-dependent pattern, mortality findings should be interpreted cautiously because the study was not powered to detect survival differences. Even so, the overall pattern is consistent with clinical observations showing that delayed source control is associated with worse outcomes [7, 16, 17]. The lung data also support the possibility that timely intervention may attenuate remote organ injury, particularly sepsis-associated acute lung injury (ARDS), which remains a major contributor to death in sepsis [7, 16–19].

Early SC sharply reduced bacterial burden in blood, peritoneum, and BAL, indicating more effective containment of systemic spread. These findings should be interpreted in the context of the standardized antibiotic regimen, as all CLP groups received meropenem starting 6 h after induction. Therefore, between-group differences likely reflect the combined effects of antimicrobial therapy and source elimination, rather than source control alone, and this may have attenuated differences between groups, particularly around the time of antibiotic initiation. Lower bacterial burden was accompanied by lower levels of several inflammatory mediators, including TNF-α, IL-6, and IL-1β, consistent with attenuation of ongoing pathogen-driven host activation.6, 19 At the same time, the cytokine response was not uniform across mediators or time points. IL-6, for example, remained detectable even with early SC, suggesting persistent immune activity despite better source control. Within the early-intervention groups, the CLP + SC-12 h group showed one of the clearest combined patterns of lower pulmonary cytokines and lower histological injury, although this was not consistent across all mediators.

The translational relevance of these findings is reinforced by human data. Martínez et al. [7] showed survival benefits of adequate SC in septic shock; Kim et al. [16] reported lower mortality when SC was achieved within 6 h; and Reitz et al. [17] demonstrated stepwise increases in mortality with progressive delay. Contemporary clinical and surgical guidance also emphasizes that SC should be both early and adequate. [19–21]. Additional evidence from Roger et al. [14], Rüddel et al. [8], and a post-hoc analysis of the AbSeS multinational cohort [22] further supports the importance of timely and adequate source control within the broader context of sepsis care. In this setting, the main contribution of the present work is not to restate the clinical value of early SC, but to describe the biological changes associated with different delays in source control under controlled experimental conditions. This may help identify which processes are most sensitive to delay and guide future mechanistic and interventional studies.

These observations are also consistent with the pathophysiology suggested by our model. The CLP model remains the gold standard for experimental polymicrobial sepsis [10, 11], and incorporating timed definitive SC enhances translational relevance by approximating source removal after established infection. Persistent abdominal infection may sustain pathogen exposure and prolong systemic cytokine drive, potentially contributing to endothelial dysfunction, altered permeability, disordered microvascular flow, and distal organ injury [6, 23]. Our physiological data fit this sequence: untreated or delayed-source-control animals showed more pronounced hypotension, lactate elevation, bacterial dissemination, and lung injury, whereas earlier SC was associated with a more favorable physiological, microbiological, and pulmonary profile; MAP recovery was most pronounced in the SC-12 h group. Our lung findings are also consistent with experimental evidence that cytokine-mediated endothelial dysfunction and innate immune activation contribute to sepsis-induced lung injury [24], and with the concept of compartmentalized immune responses in critical illness, where inflammatory activity within the infected abdominal compartment and the lung may not be fully captured by circulating markers alone[25].

At the organ level, SC reduced BAL neutrophilia across intervention groups, whereas total BAL protein did not differ significantly at 72 h. BAL protein is a widely used surrogate for alveolar-capillary permeability, but its sensitivity is limited at a single late time point: protein accumulation may peak early and partially resolve by 72 h, lavage recovery variability may obscure modest between-group differences, and the signal may persist even when cellular and cytokine responses have improved. This dissociation between BAL protein and other lung injury metrics, including neutrophilia, cytokines, and histological score, suggests that inflammatory cell trafficking and soluble mediator responses may recover earlier than structural permeability changes. More broadly, it reinforces the importance of interpreting experimental lung injury across complementary domains rather than relying on a single biomarker [26]. Recent translational work in ARDS supports this view, showing that the cellular composition of the alveolar compartment, and particularly sustained neutrophilic inflammation, is closely linked to ongoing lung injury and outcome [27].

The pulmonary cytokine profile should be interpreted cautiously, as the effect of SC was not uniform across mediators or intervention time points. Although SC was associated with broad attenuation of the pulmonary inflammatory response, the 12 h SC group showed one of the clearest combined patterns of cytokine reduction, whereas other groups displayed more heterogeneous changes. In particular, lung homogenate IL-6 was reduced in the 12 h and 18 h groups relative to CLP, but this pattern was not consistent across all time points. In supplementary exploratory pooled early-versus-delayed comparisons, pooled early SC was also associated with lower neutrophil influx, lower lung injury score, and attenuation of selected pulmonary cytokines compared with delayed SC; however, this pattern was not uniform across all mediators, and anti-inflammatory markers such as IL-10 and IL-13 were lower in some delayed-SC comparisons. Overall, these findings are consistent with the concept that prolonged uncontrolled infection may contribute to maladaptive host responses affecting both pro- and anti-inflammatory arms of the immune system, although these mechanistic inferences remain cautious [23, 28].

This study has several limitations. First, all outcomes were assessed at a single 72-h time point, so early differences in bacterial clearance, cytokine trajectories, and permeability between groups could not be captured. This endpoint was chosen to allow integrated assessment of microbiological, physiological, and histological outcomes after established sepsis, although it necessarily reduced temporal resolution. In addition, because only animals surviving to 72 h were available for biochemical and histological analyses, these endpoint data are subject to survivor bias and may underestimate the biological impact of delayed SC. Future studies should incorporate serial sampling at earlier time points (e.g., 12, 24, and 48 h) to better define the temporal evolution of infection, inflammation, and organ injury. Second, in the delayed SC groups, some animals may have died before receiving the planned intervention, further complicating the interpretation of survival and group-level comparisons. Third, the SC procedure involved a second laparotomy that was not performed in the CLP group. Although the additional surgical stress was likely modest relative to the septic insult, a sham reoperation control was not included, and a contribution of the procedure itself cannot be fully excluded. Fourth, we focused on cardiopulmonary endpoints and did not assess hepatic, renal, or neurological dysfunction. Finally, mechanistic pathways linking infection burden to endothelial and epithelial barrier dysfunction, including coagulation activation, glycocalyx shedding, and inflammasome signaling, were not evaluated, limiting mechanistic resolution. Experimental sepsis and acute lung injury literature also supports the need for multidomain assessment across time points and compartments [18, 29, 30]. In addition, this study was underpowered for certain endpoints, particularly survival, and these findings should therefore be interpreted with caution. Nevertheless, incorporating time-defined surgical source control into the CLP model enhances its clinical relevance and may provide a useful framework for investigating how delays in source control relate to remote organ injury.

## Conclusions

This study provides experimental evidence that the timing of surgical SC influences bacterial dissemination, systemic inflammation, and sepsis-associated lung injury in polymicrobial abdominal sepsis. SC within 6–12 h was associated with lower bacterial burden, improved metabolic profile, and attenuation of pulmonary inflammatory and histological injury; hemodynamic recovery was most pronounced in the SC-12 h group.

Although the survival curves showed a numerical time-dependent pattern, mortality differences were not statistically significant and should be considered exploratory. Overall, these findings reinforce the importance of source control as a time-sensitive therapeutic priority in abdominal sepsis and provide a mechanistic framework for future studies incorporating earlier endpoints, multi-organ assessment, and molecular profiling.

## Supplementary Information

Additional file 1

Additional file 2

Additional file 3

## Data Availability

The datasets generated and analyzed during the current study are available from the corresponding author on reasonable request.
