# Supplementary material for: Source control within 12 h attenuates lung injury and systemic bacterial burden in a rat model of polymicrobial abdominal sepsis (cecal ligation and puncture)
Source: Intensive Care Med Exp. 2026 Jul 17;14:94. doi: 10.1186/s40635-026-00941-1 (PMC13379536; doi:10.1186/s40635-026-00941-1)

Supplementary Figure 1. Body weight and survival data in pooled early versus delayed SC groups. (A) Body weight loss at 72 h relative to baseline body weight in pooled early SC (6 h + 12 h) and pooled delayed SC (18 h + 24 h) groups. Data are presented as mean ± SD. **(B)** Kaplan–Meier survival curves over the 72-h experimental period in pooled early SC (6 h + 12 h) and pooled delayed SC (18 h + 24 h) groups.

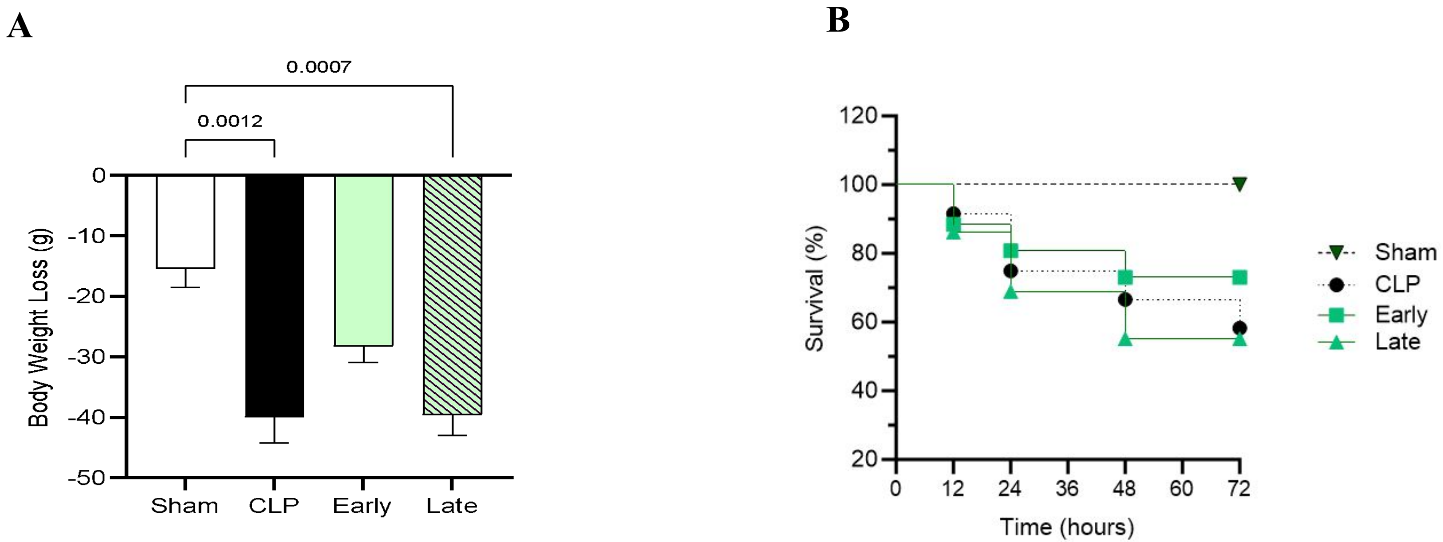

Supplement: Supplementary file 1 — Additional file 1 [file 40635_2026_941_MOESM1_ESM.pdf]
