# Supplementary material for: Source control within 12 h attenuates lung injury and systemic bacterial burden in a rat model of polymicrobial abdominal sepsis (cecal ligation and puncture)
Source: Intensive Care Med Exp. 2026 Jul 17;14:94. doi: 10.1186/s40635-026-00941-1 (PMC13379536; doi:10.1186/s40635-026-00941-1)

Supplementary Figure 2. **Additional physiological, microbiological, and plasma inflammatory data in pooled early versus delayed SC groups. (A)** Serum lactate concentration. **(B)** Oxygen saturation (SatO2). **(C)** Arterial partial pressure of carbon dioxide (PaCO2). **(D)** Blood bacterial counts. **(E)** Peritoneal bacterial counts. **(F)** Bronchoalveolar lavage (BAL) bacterial counts. **(G)** Plasma tumor necrosis factor-α (TNF-α). **(H)** Plasma interleukin-6 (IL-6). Values are presented as mean ± SD. Sample size varied by panel according to 72-h survival and sample availability. Plasma cytokine measurements were available in fewer animals in some groups because of limited sample volume.

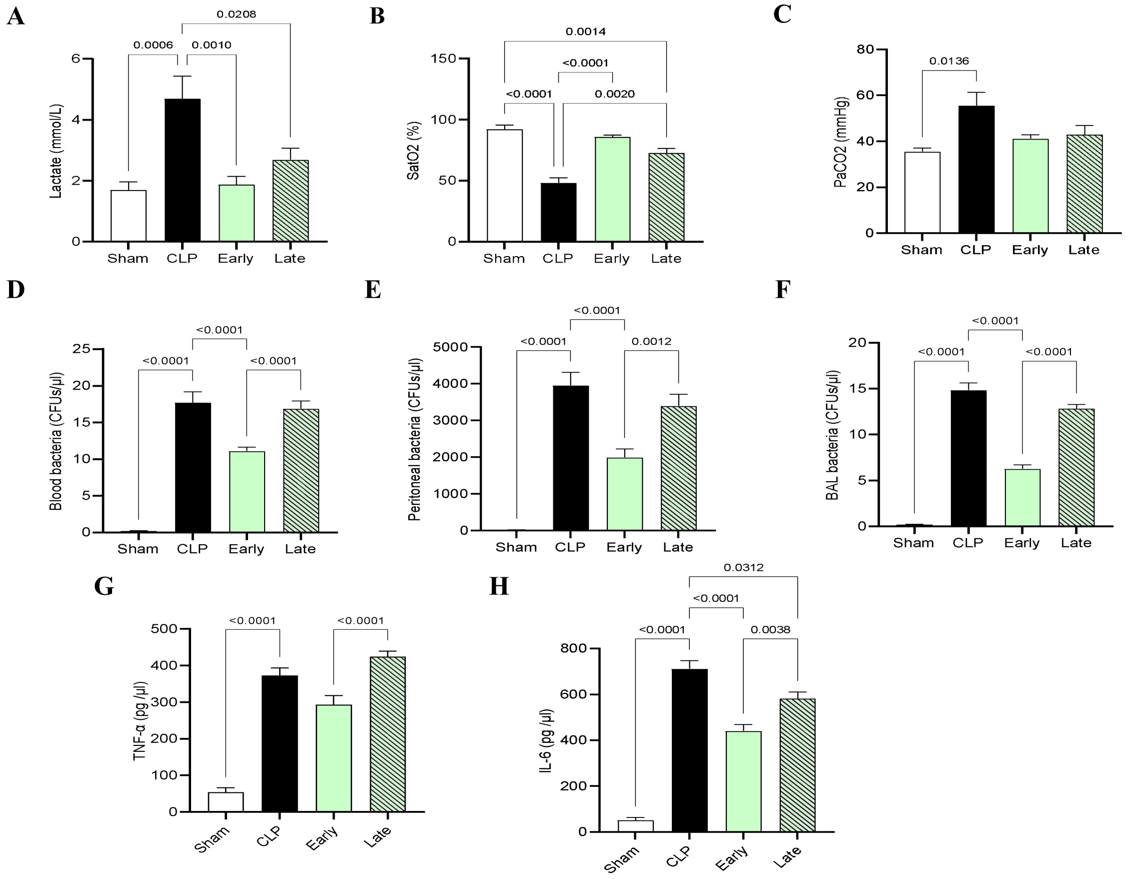

Supplement: Supplementary file 2 — Additional file 2 [file 40635_2026_941_MOESM2_ESM.pdf]
