# Supplementary material for: Source control within 12 h attenuates lung injury and systemic bacterial burden in a rat model of polymicrobial abdominal sepsis (cecal ligation and puncture)
Source: Intensive Care Med Exp. 2026 Jul 17;14:94. doi: 10.1186/s40635-026-00941-1 (PMC13379536; doi:10.1186/s40635-026-00941-1)

Supplementary Figure 3. Lung injury parameters in pooled early versus delayed SC groups. (A) Percentage of neutrophils in bronchoalveolar lavage (BAL). **(B)** Lung injury score (LIS) evaluated in H&E-stained lung sections. Lung homogenate cytokines were measured by multiplex assay: **(C)** TNF-α, **(D)** IFN-γ, **(E)** IL-1β, **(F)** IL-2, **(G)** IL-4, **(H)** GRO-α, **(I)** GM-CSF, **(J)** MCP-1, **(K)** IL-10, and **(L)** IL-13. Values are presented as mean ± SD. Sample size varied by panel according to 72-h survival and tissue availability.

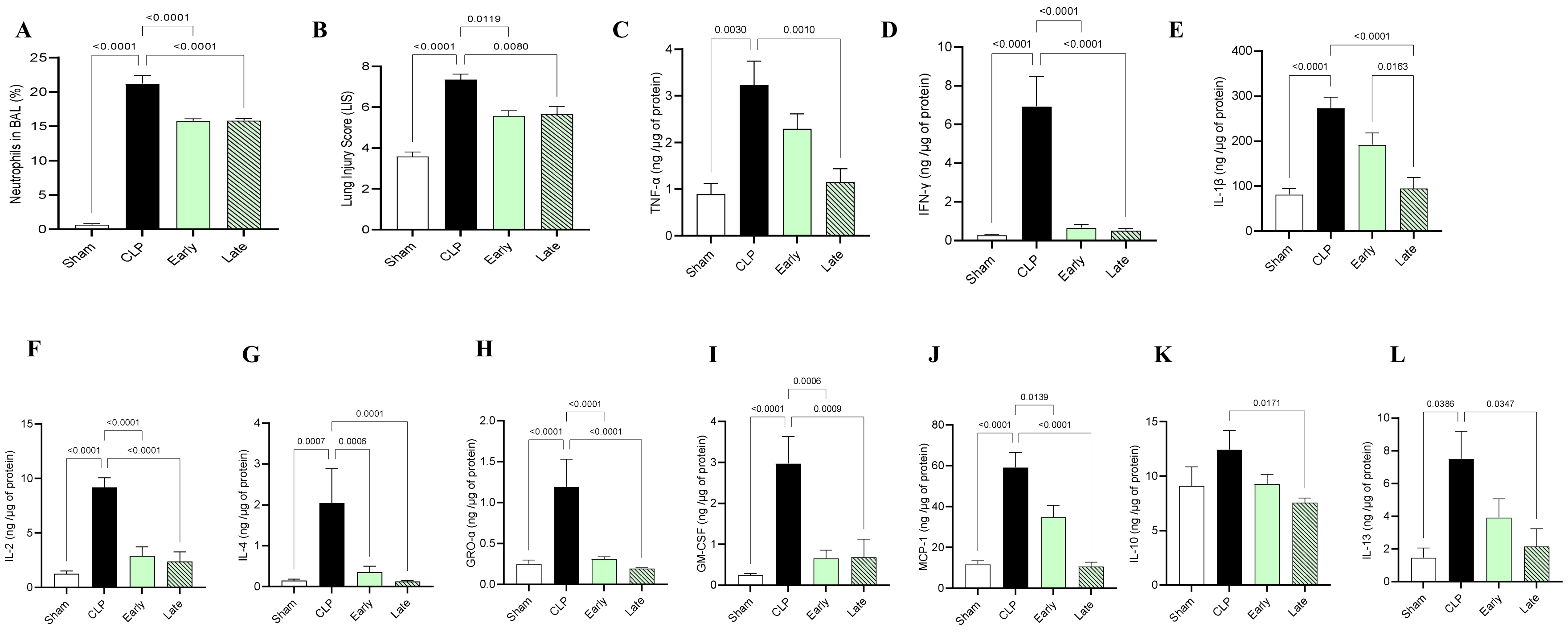

Supplement: Supplementary file 3 — Additional file 3 [file 40635_2026_941_MOESM3_ESM.pdf]
